# Effects of a positive thinking program on hope and sleep quality in Iranian patients with thalassemia: a randomized clinical trial

**DOI:** 10.1186/s40359-021-00547-0

**Published:** 2021-03-16

**Authors:** Somayeh Makaremnia, Marieh Dehghan Manshadi, Zahra Khademian

**Affiliations:** 1 School of Nursing and Midwifery, Shiraz University of Medical Sciences, Shiraz, Iran; 2 Islamic Azad University of Yazd, Yazd, Iran; 3 Community Based Psychiatric Care Research Center, School of Nursing and Midwifery, Shiraz University of Medical Sciences, Shiraz, Iran

**Keywords:** Beta-Thalassemia, Hope, Optimism, Positive psychology, Sleep quality

## Abstract

**Background:**

Thalassemia have a negative impact on the patients' psychological health and sleep quality. This study aimed to determine the effects of a positive thinking training program on hope and sleep quality of patients with thalassemia major.

**Methods:**

This randomized clinical trial was conducted on 78 patients with thalassemia major including 36 males (46.2%) and 42 females (53.8%) with a mean age of 25.56 ± 29.6 in Iran. Subjects were randomly assigned into experimental and control groups. Experimental group received 16 h training based on positive thinking materials published by Martin Seligman. Control group received only usual programs. Data were collected at baseline, as well as immediately and one month after the intervention, using Snyder’s Hope Scale and the Pittsburgh Sleep Quality Index. Data analysis was performed using SPSS Software 18.0; statistical tests included the independent T-test, the Chi-square, Mann Whitney, and Friedman test. Significance level was set at 0.05 in this study.

**Results:**

The experimental group had a significantly higher mean hope score compared to the control group immediately (45.38 ± 7.82 vs. 35.32 ± 5.54, *P* < 0.001) and one month following intervention (44.67 ± 3.47 vs. 35 ± .54, *P* < 0.001). Moreover, the mean sleep quality scores of the experimental group was significantly greater than that for control group immediately (5.35 ± 2.02 vs. 7 ± 2.4, *P* = 0.004) and one month after the intervention (4.23 ± 2.2 vs.7.02 ± 3.03, *P* < 0.001)**.**

**Conclusion:**

Since our training program on positive thinking improved hope and quality of sleep in patients with thalassemia major, we recommend the use of such courses as an important step toward promotion of hope and sleep quality among these patients.

*Trial registration* The name of the registry: Iranian Registry of Clinical Trials. Trial Registration Number: IRCT2017010431774N1. URL of the trial registry record: https://en.irct.ir/trial/24923. Registration Date: 07/03/2017.

**Supplementary Information:**

The online version contains supplementary material available at 10.1186/s40359-021-00547-0.

## Background

Thalassemia is the most common recessive genetic disorder in the world. The annual birth of individuals with beta thalassemia is estimated 22,989. In addition, it is estimated that 80 million people worldwide carry beta thalassemia and about ninety percent of annual incidence of major beta thalassemia occur in low- and middle-income countries [1]. Thalassemia has a prevalence of 10–15% in Mediterranean regions and South-Eastern Asia and 8% among the African-American community [2]. Iran is considered as one of the countries with a high prevalence of thalassemia; in this regard, more than three million carriers of beta thalassemia and approximately 20,000 patients with beta thalassemia live in Iran [3].

Thalassemia would impose a lot of burden on the patients and their families. Frequent visits to the medical center for blood transfusion leads to changes in lifestyle, inhibition of social activities, reduced self-esteem, feelings of incompetence and humiliation due to dependence on others, anger, frustration and fear of early death [4]. Research shows that patients with thalassemia are prone to socio-economic problems and psychological disorders such as anger and depression [5]. Moreover, issues related to marriage, attitude toward important events in life, occupation, economic burden, emotional concerns and medical services are among factors with a significant impact on the level of hope in people with thalassemia major. Evidence has revealed that these patients suffer a huge amount of stress, which would engage both their bodies and minds. What’s more, feeling close to death would result in a disregard for the future and lack of proper planning [6]. In one study, about 66.7% of the patients with thalassemia major had a mediocre mental image of themselves and around 37.3% were desperate to some degree [7].

Evidence indicates a significant relationship between hopefulness and mental health [8, 9]. Hope is an indicator of a person’s psychological dimensions, which relate to both mental and physical health; hope is the ability to believe in a better feeling toward the future. However, despair is the antithesis of hope and one of the distinctive features of depression [10]. Seligman considers hope as an emotional state that results in a positive attitude toward future events in life [11]. Previous studies have discussed various methods for improvement of hope in patients. In this relation, group logo-therapy has been able to increase the hope in life and improve the general health in patients with thalassemia major [12]. Furthermore, another research showed a significant difference between elderly people’s hope in life before and after group counseling [13]. In addition, an optimism-promoting program improved hope and life satisfaction among patients with heart disease [14].

Patients with thalassemia major also suffer from sleep disorders [15]. Sleep is considered as one of the basic physiological needs of the human body; however, physical and mental illness can disrupt a person’s sleep patterns [16]. In one study, sleep quality score of patients with thalassemia major was reported as 7.9 using the Pittsburgh Sleep Quality Index (PSQI), which indicates the low quality of sleep in these people [17]. Moreover, there has also been report of a poor sleep quality among 31.6% of these patients [16]. Sleep disorders and breathing problems during sleep are common issues for thalassemia patients and patients with sickle cell disease [18]. This sleep disorder could be a result of the short-time and frequent hospitalization periods these patients have to go through. On the other hand, various studies have shown that patients with thalassemia major suffer from anxiety and depression, and the symptoms caused by these disorders can have an influence on their sleep quality [19]. Furthermore, research has revealed an inverse relationship between sleep quality and anxiety in patients with thalassemia major; meaning that an increased anxiety would result in a reduced quality of sleep [20].

Previous studies have shown the effectiveness of positive psychotherapy on psychological health indicators of individuals with chronic conditions [21–24]. In Martin Seligman's perspective, positive thinking means to focus on human capabilities, such as the ability to live happily, enjoy, solve problems and be optimistic, rather than the shortcomings and weaknesses [25]. In positive thinking, subjects such as hope, wisdom, creativity, bravery, spirituality, responsibility and perseverance are discussed more [26]. A research on female patients with Multiple Sclerosis has revealed that positive thinking can increase individuals' sleep quality and reduce their death anxiety [23]. In another study on female patients with thalassemia major, a 10-h positive thinking training program improved patients' happiness and decreased their perceived stress [21].

As mentioned earlier, patients with thalassemia major suffer various psychological disorders, such as anger, depression and anxiety, which would all affect the sleep quality and levels of hope. A review of literature in the field reveals few studies worldwide relating hope and sleep quality in thalassemia patients. On the other hand, the effectiveness of positive psychology interventions on psychological health of patients with chronic conditions has been shown. However, we did not encounter any research on the impact of positive thinking interventions on sleep quality and hope in patients with thalassemia. Because of the unique nature of thalassemia disease and the patients' psychological health, the studies on patients with other chronic diseases may not be applicable to these patients. To provide evidence-based nursing services, more research is needed to investigate the effectiveness of interventions on the sleep quality and hope among these patients with thalassemia. Therefore, we decided to conduct the current study aiming to determine the effect of positive thinking training program on sleep quality and hope in patients with beta thalassemia major. We hypothesized that such programs could improve the hope and sleep quality among patients with thalassemia major.

## Methods

### Design

This was a randomized clinical trial. Data were collected by a trained assistant from July to December 2017. The study adheres to CONSORT guidelines (Additional File 1).

### Study settings

The study was performed in Shariati hospital, Fars province, in southwestern Iran. The hospital is the referral site for patients with thalassemia.

### Samples

Study sample consisted of all patients with beta thalassemia meeting the inclusion criteria (n = 78), who then entered the study through census. Inclusion criteria were the minimum age of 18 years old and literacy, willingness to participate, ability to complete self-rating questionnaires, not substance use, no membership in other positive thinking groups, having no diagnosis of psychiatric disorders such as depression treated with medication (based on the patients’ medical chart information) and not being in a major crisis, such as the death of a close relative, during the course of study or the previous three months. Exclusion criteria included failure to answer more than 20% of the questionnaires' items, and not attend four or more training sessions. Compensatory sessions were held for those who were absent for a maximum of three training sessions.

Participants were equally assigned into experimental and control groups (39 members each) by permuted block randomization. At the first, the statistician prepared the order of 19 random blocks of size 4. In this way, firstly, it was randomly determined that A represented the experimental group and B represented the control group. Possible states of the blocks were AABB, ABAB, ABBA, BAAB, BBAA, and BABA, each was given a numeric code from 0 to 5, respectively. Then, using a table of random numbers, the starting point was randomly selected, followed by 18 numbers in the rows. By considering the order of the numbers in the table, the permutation for each number was determined. For example, if the first three numbers were 1, 0 and 5, respectively, the order of the first 12 participants of the patients' list was ABABAABBBABA. Therefore, by selecting 19 numbers from the table, the method of allocating a total of 78 people to the two groups was determined.

**Fig. 1:**
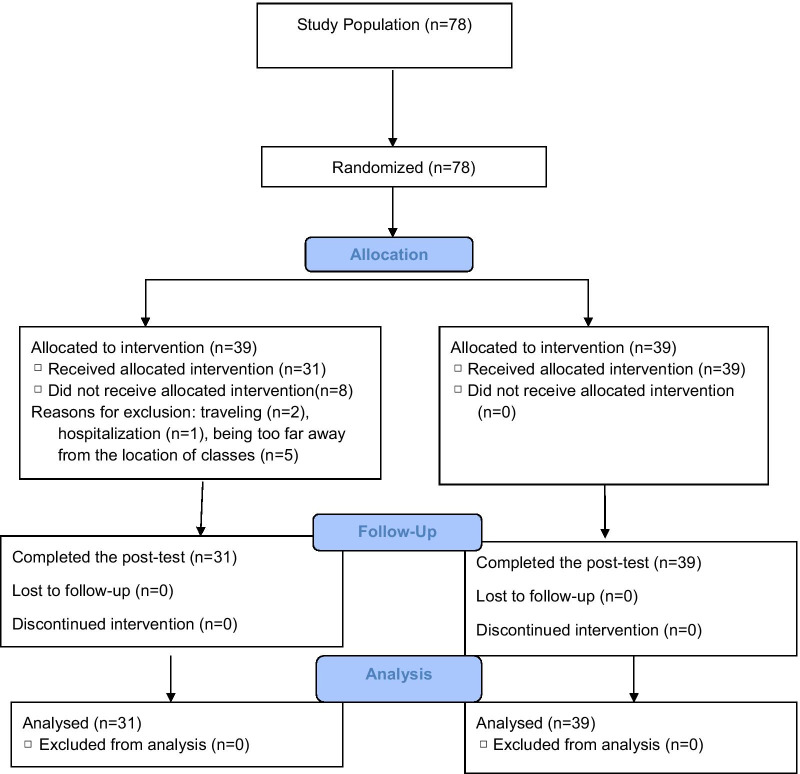
CONSORT flow diagram of the randomized controlled trial. The diagram shows progress of participants throughout the study. After random allocation of patients to the experimental and control groups, eight patients in the experimental group did not receive the allocated intervention. Finally, data from 31 individuals in the experimental group and 39 individuals in the control group were analyzed

There were eight cases of exclusion in the experimental group. Seven patients did not attend in the training program; two patients as a result of traveling and five individuals because of being away from the location of classes could not participate in the training program. Moreover, one participant was excluded due to hospitalization. Finally, 31 patients remained in the experimental group and 39 individuals remained in the control group (Fig. 1).

### Intervention

Intervention involved a positive thinking training program executed in eight two-hours sessions held twice per week. Educational materials were based on the positive psychotherapy published by Seligman in his books [27, 28]. Furthermore, the seventh session focused on the methods of anger management based on the patients’ needs. Sessions were conducted by one of the researchers and a psychologist. Table 1 presents the general contents discussed in each session. Educational methods included lecture, question and answer and educational video clips and audio files based on the contents of each session. Participants were also assigned practice at the end of each session, which they did personally at home; previous assignments were discussed at the beginning of each session.

**Table 1:** Positive thinking program

1st Session	Explanation of procedures and reasons for our specific selections, introduction to the concept of positive thinking, group introductions and a review of rules
2nd Session	Factors affecting health, familiarity with changeable and unchangeable elements in life
3rd Session	Steps to accepting the unchangeable conditions of life, ways to deal with unchangeable conditions in life
4th Session	Ways to overcome depression, specification of values and goals in life
5th Session	Assessment of satisfaction with life and the ability to live happily, being positive by challenging negative thoughts, use of productive language and reconsideration in beliefs
6th Session	Anger Management
7th Session	Connecting with the present time through mindfulness meditation
8th Session	Experiencing the present time through mindfulness and a recapitulation of contents presented during the course

Data collection and intervention program were mostly performed while patients were waiting to receive hemoglobin test results, so that no additional referrals to the hospital were required. Control group did not receive any intervention. They received only usual programs including watching TV and reading magazines during presence in the hospital. To prevent contact between patients, the patients of two groups were in the hospital on different days, with the coordination of the hospital staff. In addition, the experimental group was urged not to talk to other patients about the educational content during the study. At the end of the study, the control group was given the positive thinking training program on the Compact Disk.

### Measures

Patients completed self-report questionnaires individually, before, immediately after and one month after intervention in the conference room of the hospital. Data collection tools included a demographic information form, Snyder’s Hope Scale and the PSQI.

The demographics consisted of general information including age, gender, marital status, education and place of residence.

Snyder’s hope scale comprises 12 items, rated based on 4-point Likert scale from strongly disagree (1) to strongly agree (4); items 3, 7 and 11 were rated inversely strongly disagree (4) to strongly agree (1). Items 3, 5, 7 and 11 are related to distract and are not scored. In this questionnaire, a higher score indicates a higher level of hope in the respondent; scores ranges between 8–32. Snyder et al. reported the confirmation of the scale validity by factor analysis that indicated two components of agency and pathway. Moreover, for convergent and discriminant validity, they reported the scale's positive and negative correlation with other scales. For instance, they reported the scale's correlation with Rosenberg Self-Esteem Scale (r = 0.58, *P* < 0.005) and depression scale (r = -0.60, *P* < 0.001). Moreover, they reported its internal consistency by Cronbach's alphas ranging from 0.74 to 0.84, and its stability by test–retest correlations of 0.73 to 0.85 [29].

In the study by Khodarahimi, using factor analysis and principal component analysis, the validity of the scale’s Persian version was determined as 133.48 (*df* = 66), based in Bartlett’s Test (*P* = 0.0001). The scale’s reliability was verified with a Cronbach’s alpha equaling 0.90 [30].

The PSQI was designed by Buysse et al. to measure the quality of sleep and help detect individuals with good or poor sleep quality. This questionnaire includes 18 items, each rated based on a scale ranging from 0 to 3, 3 indicating poor sleep and 0 meaning a very high sleep quality. The questionnaire’s overall score ranges between 0 and 21. In this regard, the sum total of all poor scores would yield the overall score for sleep quality; therefore, scores over 5 suggest poor sleep and severe problems in at least two areas or average problems in more than 3 areas, which indicate poor sleep quality. This index covers seven dimensions, including "subjective sleep quality", "sleep latency", "sleep duration", "sleep efficiency", "sleep disturbances", "use of sleep medication" and "daytime dysfunction". To confirm its validity, Buysse et al. showed that the instrument discriminated patients with control group and its findings were somewhat comparable with polysomnographic findings. Furthermore, they confirmed its reliability by internal consistency of 0.83 and test–retest stability of 0.85 [31]. Mohammad Gholi Mezerji et al. (2013), confirmed the validity of Persian version of the index was based on content validity index ≥ 0.90, and factor analysis. Its reliability was verified by Cronbach’s alpha of 0.65 [32].

### Data analysis

Data analysis was performed via SPSS Statistics 18.0 using descriptive and analytical tests. The Kolmogorov–Smirnov test was used to assess the normality of data distribution. For inter-group comparisons based on qualitative variables, the Chi-square test was employed. Moreover, independent T-test was used to compare groups based on age, and the Mann–Whitney U test was used to compare other quantitative variables between groups.

In order to evaluate the changes in a quantitative variable during the course of time, we used the Friedman test. To prevent time-group interaction, we split data of sleep quality and hope based on groups; then Friedman test was performed in each group separately.

According to the exclusion criteria, the patients who did not attend four or more training sessions should be excluded from the study. The excluded patients did not attend any training sessions; therefore, their data were not included in data analysis, and we did not perform intention to treat analysis [33].

The Cohen's d effect size for paired T-test was calculated. The effect sizes of 0.8, 0.5, and 0.2 were considered large, medium, and small, respectively [34]. *P* value less than 0.05 was considered statistically significant.

## Results

In this research, participants had a mean age of 25.5 ± 6.9 (18–42); the majority of subjects were female (53.8%), single (76.9%) and unemployed (60.3%), had a high school diploma (39%) and lived in the cities (64.1%). There weren’t any significant differences between the two groups in terms of age (25.8 ± 6.5 for experimental group vs. 25.4 ± 5.9 for control group, *P* = 0.7), and other demographic variables (Table 2).

**Table 2:** The comparison of demographic variables between the experimental and control groups

Group Variable	Total	Experimental (n = 31)	Control (n = 39)	*P* value*
	n(%)	n(%)	n(%)	
Sex				
Male	32(45.7)	16(51.6)	16(41)	0.377
Female	38(54.3)	15(48.4)	23(59)	
Job				
Employed	16(22.9)	7(22.6)	9(23.1)	0.679
Housewife	12(17.1)	4(12.9)	8(20.5)	
Unemployed	42(60.0)	20(64.5)	22(56.4)	
Marital status				
Married	13(19.1)	6(33.3)	10(26.3)	0.089
Single	55(80.9)	27(90)	28(73.7)	
Education				
Subdomain	21(30.4)	8(25.8)	13(34.2)	0.099
Diploma	26(37.7)	9(29.0)	17(44.7)	
Higher than diploma	22 (31.9)	14 (45.2.8)	8(21.1)	
Location of residence				
City	43(62.3)	22(71.0)	21(55.3)	0.181
Village	26(37.7)	9(29.0)	17(44.7)	

^*^Chi Square test

A comparison of hope scores between the experimental and control groups before, immediately after and one month after intervention revealed no significant differences at baseline; however, the experimental group had a significantly higher mean hope score compared to the control group immediately (d = 1.29, Power = 1, *P* < 0.001) and one month following intervention (d = 1.68, Power = 1, *P* < 0.001). Furthermore, a comparison of mean hope scores in the experimental group at the three evaluation stages as well showed a significant increase (*P* < 0.001). However, the mean hope scores of the control group decreased over time (Table 3).

**Table 3:** Inter-group and intra-group comparison of sleep quality scores of patients with thalassemia major

Time		Before intervention		Immediately after intervention		One month after the intervention		*P* value*
Variable	Group	Mean	SD	Mean	SD	Mean	SD	
Hope	Experimental	21.1	4.4	26.7	5.8	26.2	2.0	< 0.001
	Control	21.1	5.1	20.4	3.7	20.3	4.5	0.042
	*P* value**	0.986		< 0.001***		< 0.001***		

^*^Friedman Test

**Mann–Whitney U Test

*** Large Effect size

Regarding the mean overall score for sleep quality before, immediately after and one month after intervention, the reduced scores of the experimental group indicates the improvement of sleep quality, which was also statistically significant (*P* < 0.001). However, these changes of sleep quality score were not statistically significant in the control group (*P* = 0.613). Furthermore, there were a significant reduction in the mean scores for the dimensions of "subjective sleep quality", "sleep latency", "sleep duration", "sleep disturbances" and "daytime dysfunction" one month following intervention comparing to baseline (*P* < 0.001). Meanwhile, the "use of sleep medication" did not undergo any significant changes after training. In the control group, none of the dimensions had any significant changes immediately and one month after the intervention (Table 4).

**Table 4:** Inter-group and intra-group comparison of sleep quality scores of patients with thalassemia major

Time		Before intervention		Immediately after intervention		One month after the intervention		*P*-value*
Variable	Group	Mean	SD	Mean	SD	Mean	SD	
Total sleep quality	Experimental	8.7	3.7	5.3	2.0	4.2	2.1	< 0.001
	Control	7.2	3.0	6.9	2.3	7.0	3.3	0.726
	*P* value**	0.084		0.004***		< 0.001****		
Subjective sleep quality	Experimental	1.6	0.84	1.03	0.4	0.70	0.46	< 0.001
	Control	1.25	0.71	1.17	0.5	1.17	0.5	0.545
	*P* value**	0.063		0.225		0.001		
Sleep latency	Experimental	2.0	1.0	1.38	0.88	1.12	0.76	< 0.001
	Control	1.61	0.81	1.74	0.71	1.84	0.9	0.183
	*P* value**	0.066		0.109		0.001		
Sleep duration	Experimental	0.96	1.07	0.41	0.56	0.32	0.59	0.001
	Control	0.51	0.82	0.51	0.75	0.76	0.9	0.155
	*P* value**	0.117		0.831		0.026		
Sleep efficiency	Experimental	0.77	1.14	0.45	0.72	0.19	0.47	0.046
	Control	0.92	1.15	0.64	0.90	0.79	0.97	0.326
	*P* value**	0.483		0.457		0.002		
Sleep disturbances	Experimental	1.54	0.50	1.09	0.30	1.06	0.35	< 0.001
	Control	1.50	0.55	1.5	0.50	1.44	0.60	0.850
	*P* value**	0.623		0.001		0.001		
Use of sleep medication	Experimental	0.29	0.73	0.09	0.30	0.19	0.60	0.076
	Control	0.3	0.76	0.15	0.43	0.28	0.6	0.206
	*P* value**	0.864		0.660		0.402		
Daytime dysfunction	Experimental	1.51	0.81	0.87	0.49	0.64	0.66	< 0.001
	Control	0.89	0.71	1.15	0.53	0.97	0.62	0.104
	*P* value**	0.003		0.029		0.019		

^*^Friedman Test

^**^Mann–Whitney U Test

^***^Medium effect size

^****^Large effect size

A significant difference was observed between the two groups’ mean sleep quality scores immediately (d = 0.74, Power = 0.91, *P* = 0.004) and one month after intervention (d = 1.01, Power = 0.99, *P* < 0.001). In addition, the experimental group had lower mean scores than the control group on dimensions of "sleep disturbances" and "daytime dysfunction", both immediately and one month after the intervention (*P* < 0. 05). In addition, the experimental group mean scores on "subjective sleep quality", "sleep latency", "sleep duration", and "sleep efficiency" were significantly lower than the mean scores of the control group one month after the intervention. However, the between group comparison of the mean scores of "use of sleep medication" dimension were not significant immediately, nor one month after the intervention (Table 4).

## Discussion

Our results showed improvements in both sleep quality and hope of the participants after intervention based on Martin Seligman's positive psychology. The effect size values of the changes indicate that these improvements are clinically important. Based on the findings, our hypothesis the positive thinking training program is effective on the hope and sleep quality of patients with thalassemia major is confirmed.

Although there are limited studies discussing the influence of positive thinking training programs on sleep quality and hope in patients with thalassemia, a number of studies have demonstrated the effectiveness of positive thinking interventions. A positive thinking training program improved happiness and reduced stress among Iranian patients with beta thalassemia. In addition to the differences in the dependent variables, the mentioned study had other differences from the present study, including that it used a smaller sample size than this study and was performed only on women [21]. In another study on patients with Parkinson’s disease the desirable impact of positive thinking on optimism and their understanding of disease was reported [35]. Moreover, one meta-analysis showed the positive effect of positive psychotherapy interventions on improved mental health and psychological well-being, as well as reduced signs of depression [36]. In a research by Farnam and Hamidi, a group positive thinking training program with an emphasis on the Quran and Islamic teachings was able to increase hope among employees of the Department of Education; these findings were still consistent after two months [37]. Furthermore, ducational nursing interventions is effective in improving quality of life of patients with chronic diseases [38].

A number of studies have demonstrated the positive impact of other interventions on patients' hope levels. In this regard, a research by Gholami et al. showed that group logo-therapy can increase the hope in patients with thalassemia major; their follow-up one month after intervention revealed the same results [12]. Another research reported a significant difference between the hope levels of elderly people before and after group counseling [13]. The positive thinking program in the current study emphasized the patients’ positive perspective toward themselves and others, and acceptance of the disease and the unchangeable conditions of life. In addition, emphasis was placed on specification of values and short-term goals and practice of mindfulness meditation with the aim to increase attention to the present. It seems that these emphases have been able to stimulate patients to plan for the future and improve their levels of hope.

Regarding sleep quality as well, previous studies have demonstrated the therapeutic effects of various interventions. For instance, in a research by Elahiyan Borojeni et al., movie-based education improved the overall scores for sleep quality and the dimensions of subjective sleep quality, sleep disorders and sleep latency in patients with bronchial asthma [39]. Another study suggested the desirable effect of sleep health education on quality of sleep among nurses [40]. Another study introduced a multi-components web application to improve sleep quality and to decrease stress among college students [41]. Contrary to our findings, lavender aromatherapy did not have any effects on the sleep quality of hemodialysis patients; this study was performed among 60 patients on hemodialysis; thus, it was different from the present study both in terms of sample and type of intervention. It seems that the continued practice of positive thinking skills taught in our study, such as mindfulness meditation before sleeping hours, has been successful in maintaining the effects of the intervention on sleep quality in the one-month follow-up period after training.

### Clinical implications

Based on the findings of this research, we can conclude that eight sessions of positive thinking training and familiarization with positive psychotherapy can improve the levels of hope and sleep quality in patients with thalassemia major.

## Research limitations

In this study, the researcher had access to a rather small statistical population; nevertheless, all patients who met the inclusion criteria entered the study through census. Another limitation is that the attrition occurred only in the experimental group. Nevertheless, the data analysis showed a good power for our results. This can improve the reliability of the study.

The researchers used measures such as patients from both groups coming to the hospital on different days and emphasizing not talking about the training program with other patients to reduce contact between patients in both groups. However, the possibility of information leakage between the two groups exists. Furthermore, due to the nature of our intervention, it was not possible to blind the participants, but the researcher was blinded and an assistant collected the required data.

## Conclusion

Based on the findings of this research, we can conclude that eight sessions of positive thinking training and familiarization with positive psychotherapy can improve the levels of hope and sleep quality in patients with thalassemia major. It seems that positive thinking can remove negative thoughts and despair from the mind and be effective by reminding a person of the positive aspects of life, improving the relationships with others and the self and teaching strategies to overcome the stress and anxiety brought about by disease. Since nurses can play an influential role in prevention and management of complications in patients with thalassemia major, they need to pay full attention to the patients’ hopefulness and quality of sleep as well. Therefore, it is necessary that nurses first learn the positive thinking skills themselves, and then integrate them in the patients’ care protocols. What’s more, with further research, we can use positive thinking programs as a convenient, inexpensive and non-drug intervention adjuvant to psychology and nursing interventions in order to manage the sleeping problems and hope levels among patients with thalassemia major. We recommend further research on the subject with larger sample sizes and longer follow-up.

## Supplementary Information

**Additional file 1**. CONSORT 2010 checklist of information to include when reporting a randomised trial.

## Data Availability

The dataset of the present study is available upon request.

## Abbreviations

SPSS: Statistical Package for the Social Sciences
ANOVA: Analysis of variance
PSQI: Pittsburgh Sleep Quality Index
